# Integrating Artificial Intelligence Into Exposure Therapy: A One Year Follow‐Up Case Report of Emetophobia With Comorbid Panic Disorder

**DOI:** 10.1002/ccr3.71715

**Published:** 2026-01-16

**Authors:** U. Selen Kilic

**Affiliations:** ^1^ Private Practice Istanbul Türkiye

**Keywords:** artificial intelligence, ChatGPT, cognitive behavioral therapy, emetophobia, exposure therapy, panic disorder

## Abstract

Emetophobia is a specific phobia characterized by an intense fear of vomiting, often accompanied by panic attacks, hypervigilance to bodily sensations, and avoidance behaviors. This case study describes the Cognitive Behavioral Therapy (CBT)‐based treatment of a 24‐year old woman with Emetophobia and comorbid panic disorder. Her symptoms caused significant impairment, leading her to avoid social settings, public spaces, and situations linked to vomiting (e.g., dining out, public transport, certain media content). Treatment followed a structured CBT protocol for specific phobias, including psychoeducation, cognitive restructuring, exposure therapy, response prevention and attention‐shifting techniques. A novel feature of the intervention was the inclusion of brief, spoken interactions in English with a speech‐based artificial intelligence tool ChatGPT. As English was not the client's first language, these interactions required increased cognitive effort, serving as a structured external distraction that facilitated habituation by helping her remain in anxiety‐provoking situations without relying on safety behaviors. Treatment progress was tracked using validated self‐report measures including the Specific Phobia of Vomiting Inventory, the Emetophobia Questionnaire‐13 and Panic Disorder Severity Scale. Substantial symptom reduction was observed across domains, with improvements maintained at one‐year follow‐up. By the end of therapy, the client no longer depended on safety behaviors and demonstrated greater tolerance for bodily sensations and uncertainty. This case highlights the potential of CBT for treating Emetophobia and suggests that the integration of artificial intelligence, when applied with clear clinical rationale, may improve attentional flexibility and enhance treatment engagement.

## Key Clinical Message

This case highlights how integrating artificial intelligence into exposure therapy can enhance outcomes for Emetophobia with comorbid panic disorder. AI assisted attention‐shifting supported sustained engagement, reduced avoidance, and maintained clinical gains at one‐year follow‐up, demonstrating the potential of blending digital tools with CBT to strengthen traditional therapeutic approaches.

## 1 Introduction

Emetophobia, fear of vomiting and nausea, or “Specific Phobia–Other (Vomiting)” in the *Diagnostic and Statistical Manual of Mental Disorders*, *Fifth Edition (DSM‐5)* [1]. This specific phobia includes fears related to vomiting in public, witnessing others vomit, observing the act itself, or even experiencing nausea. Its symptoms can manifest across cognitive, emotional, and physical domains. Individuals with Emetophobia often experience disruptions in social, academic, and occupational functioning, along with significant limitations in leisure and daily activities [2]. Most studies have focused on adult populations and existing data suggest that it is more prevalent in females (6%–7%) than males (1.7%–3.1%) [3, 4]. Emetophobia symptoms are poorly understood [5] and there is limited research attention which might hinder the development of tailored assessment tools and treatment protocols. Research indicates that Emetophobia frequently co‐occurs with other psychiatric conditions. Among these, panic disorder is particularly prominent [6]. According to the DSM‐5, panic disorder is characterized by recurrent and unexpected panic attacks, followed by at least one month of persistent worry about additional attacks or their consequences, along with maladaptive behavioral changes such as avoidance of situations perceived as potential triggers [1]. In addition to panic disorder, individuals with Emetophobia have also been found to meet criteria for obsessive‐compulsive disorder, generalized anxiety disorder, and dysthymia, while social anxiety disorder, post‐traumatic stress disorder, and major depressive disorder have been observed at lower rates [6].

As with various anxiety disorders, the treatment of Emetophobia commonly involves exposure‐based interventions, Cognitive Behavioral Therapy (CBT), pharmacological options, or a combination of these methods [3, 7]. Boschen proposed a CBT model of Emetophobia, highlighting factors such as anxiety sensitivity, heightened focus on bodily sensations, catastrophic thinking, avoidance of nausea, negative beliefs about vomiting, somatization of gastrointestinal symptoms, and cognitive biases [5]. Maack et al. provided prior evidence supporting the CBT conceptualization of Emetophobia and the effectiveness of exposure‐based interventions, with therapeutic gains maintained at a three‐year follow‐up [8]. This finding is consistent with results from Riddle‐Walker et al., who conducted the first randomized controlled trial evaluating a CBT protocol specifically tailored for Specific Phobia of Vomiting and found that 50% of participants in the CBT group achieved clinically significant improvement [9].

With increasing technological advancement, psychotherapists are increasingly exploring integrative practices that incorporate emerging tools into established treatment models. While Virtual Reality (VR) has already demonstrated efficacy as an adjunct to exposure‐based therapies for anxiety‐related disorders [10], recent developments in artificial intelligence (AI) have opened new avenues for digital augmentation of psychotherapy. ChatGPT, a language‐based AI developed by OpenAI, has attracted global attention for its human‐like dialogue generation capabilities. Despite its popularity, empirical studies examining its clinical application remain scarce [11]. This case study contributes to the emerging literature by presenting the integration of AI into CBT, demonstrating its potential to support attention shifting and enhance engagement during exposure‐based interventions.

## 2 Case History/Examination

### 2.1 Participant

“Lara” (pseudonym) is a 24‐year‐old single Turkish woman and university student living independently. She was referred to the clinic by her psychiatrist in December 2023 for the treatment of panic disorder and specific phobia‐other type (Emetophobia), both diagnosed according to DSM‐5 criteria, with sessions conducted at a frequency of once per week. Lara was raised in a high‐anxiety family with illness‐focused maternal discourse and rigid paternal parenting. Family relationships were moderately close, with continued financial support during university. Development was within normal limits. She had minimal vomiting history, with the last episode at age 20. Previous psychodynamic therapy and EMDR following diagnosis of Emetophobia and panic disorder yielded limited benefit.

As shown in Figure S1, benign bodily sensations escalated into panic symptoms, with 8–10 weekly attacks at intake and marked functional impairment with avoidance and safety behaviors.

### 2.2 Therapist

The author of this case report is a clinical psychologist with a master's degree in clinical psychology. She is a Beck Institute Certified CBT practitioner and currently runs her own private practice in Istanbul. Throughout the treatment, the therapeutic alliance remained strong, facilitating trust, collaboration, and adherence to therapeutic tasks.

## 3 Differential Diagnosis, Investigations and Treatment

The current case study used a within‐subject design to evaluate the effectiveness of CBT integrated with AI‐assisted attention‐shifting strategies for the treatment of Emetophobia comorbid with panic disorder. The presence of comorbid panic disorder is clinically significant, as Lara experienced panic attacks not only in vomiting‐related situations but also in contexts unrelated to Emetophobic cues. This indicates that her symptoms extended beyond a specific phobia and met the broader criteria for panic disorder. As shown in Figure S1, cognitions such as “I won't find a way out,” “I will be trapped,” and “I won't be able to get home,” along with catastrophic images and focus on heart rate, were sufficient to trigger panic attacks on their own. Although the patient met criteria for both specific phobia and panic disorder, Emetophobia was the primary diagnosis due to its central role in avoidance, safety behaviors, and functional impairment, while panic disorder was considered comorbid because of unexpected attacks beyond vomiting‐related contexts. Their interaction can be understood as bidirectional: vomiting‐related cues trigger panic symptoms, while panic‐related physiological arousal (e.g., increased heart rate, sweating, dizziness) intensifies vomiting‐related fears, reinforcing the Emetophobic response.

The overall treatment flow is summarized in Figure S2. In the initial phase (Sessions 1–5), therapy began with psychoeducation aimed at increasing the client's understanding of vomiting‐related fears, physiological mechanisms, and the distinction between discomfort and actual threat. Scientific readings were shared to normalize the experience, and the client was assigned brief, but regular homework aimed at consolidating in‐session gains. Emotional responses and automatic thoughts were explored, with an emphasis on identifying emotionally charged “hot” cognitions. To enhance motivation and engagement, the client completed an imagery exercise envisioning life without Emetophobia.

The second phase (Sessions 6–10) focused on cognitive restructuring. Common cognitive distortions, including catastrophizing, over generalizing black‐and‐white thinking, were introduced. The client was supported in identifying and challenging her own maladaptive thoughts through Socratic questioning and reframing techniques. Personalized coping cards were developed to reinforce adaptive thinking. This phase also emphasized identifying the client's personal strengths and resilience, shifting the client's perspective from fear‐driven interpretations to more balanced thoughts.

The third phase (Sessions 11–33) focused on exposure and response prevention. To address comorbid panic symptoms, interoceptive exposure (e.g., spinning, breath‐holding) and cognitive restructuring were applied to reduce catastrophic misinterpretations of bodily sensations, leading to decreased hypervigilance and avoidance. Academic stress required a temporary shift in focus between Sessions 17–21, which extended the exposure schedule. Exposure was implemented through verbal, video‐based, imaginal, and in vivo tasks following a collaboratively developed hierarchy (see Table S1). Sessions included graded video exposures sourced from https://quietmindsolutions.com/video‐exposures/, which were practiced five times per session and collaboratively assigned as homework to be repeated five times daily, four days per week, to support habituation and reduce avoidance. Progression to the next step occurred when Lara's subjective anxiety consistently fell below 40%. In vivo exposures were completed as between‐session homework and were preceded by in‐session cognitive restructuring related to the anticipated exposure.

Response prevention was implemented by encouraging the client to refrain from safety behaviors (e.g., carrying antiemetics, bodily checking, threat rehearsal) and to remain in anxiety‐provoking situations to allow habituation. To support attentional flexibility and reduce internal threat monitoring during these exposures, a range of attention‐shifting strategies were introduced. These included external focus techniques such as the 5‐4‐3‐2‐1 grounding exercise, which engages multiple sensory modalities (e.g., sight, sound, touch), and sensory grounding methods like drinking cold water or touching textured objects. Mental tasks were also employed, such as backward counting by sevens (e.g., 100, 93, 86…) to increase cognitive load and redirect focus. However, during high‐intensity real‐world exposures, these techniques were often insufficient to sustain engagement and prevent avoidance, leading to the introduction of a novel intervention.

An innovative addition to treatment was the introduction of AI‐assisted verbal engagement as a structured attention‐shifting strategy during high‐anxiety exposures. Starting in Session 24, Lara engaged in voice‐based conversations with ChatGPT‐4o during high‐anxiety exposures, following a 15‐min psychoeducation segment conducted prior to implementation, which clarified its purpose, scope, and practical boundaries as a structured, time‐limited aid to prevent it from functioning as a safety behavior. This clarification was essential to prevent the tool from becoming a safety behavior that could interfere with exposure and habituation. The client reviewed relevant ethical information, was encouraged to ask questions, and provided informed consent with the option to withdraw at any time. The AI conversations, conducted in English as a non‐native language, increased cognitive demand and consisted of brief 7‐min general knowledge tasks to redirect attention from internal sensations and automatic thoughts. Each session began with a brief greeting and check‐in (e.g., “Hi, how are you today?”), followed by the client's explicit request: “Could you ask me some general knowledge questions to help distract me?” Instead of giving one‐word answers, the client responded using full sentences (e.g., “I think Mercury is the closest planet to the Sun”), which facilitated deeper cognitive engagement. She reported that her anxiety diminished significantly within seconds during these conversations, allowing her to remain focused and engaged in exposure tasks for longer periods. ChatGPT‐4o did not replace clinical intuition but complemented it by sustaining engagement during exposure exercises and reducing avoidance alongside cognitive restructuring and response prevention. By the end of treatment, the client showed marked symptom and functional improvement and viewed ChatGPT‐4o as a temporary, structured aid limited to exposure contexts that did not interfere with the therapeutic relationship.

Following the completion of active treatment (Session 33), the client entered a follow‐up phase, which included booster sessions at 1st month, 3rd month, 6th month and 12th month intervals. These sessions focused on reviewing symptom stability, reinforcing the use of cognitive and behavioral tools, and encouraging ongoing low and middle intensity exposure practices.

## 4 Conclusions and Results (Outcome and Follow‐Up)

Lara completed a battery of self‐report measures at baseline, throughout the treatment process, and during follow‐up sessions, focusing on symptom severity and treatment outcomes related to Emetophobia and panic disorder.

Symptom severity and treatment outcomes were assessed using the SPOVI [12], EmetQ‐13 [13], Panic Disorder Severity Scale (PDSS) [14]. On the SPOVI, Lara's baseline score was 52, indicating high symptom severity, which decreased to 9 at one‐year follow‐up, falling below the clinical cut‐off. On the EmetQ‐13, her baseline score was 57, reflecting severe distress, which decreased to 17 at follow‐up, indicating remission to a subclinical range. On the PDSS, her score decreased to 5 at follow‐up, falling below the clinical threshold for panic symptoms. The Turkish version of the scale, validated by Monkul et al. [15], was used in this study.

Lara's self‐rating of outcome measures is shown in Figure S3. Beyond standardized measures, Lara reported notable functional improvements in daily life. The exposure items addressed in therapy also had a positive impact on other areas that the client had previously avoided. She described being able to engage in activities she had previously avoided, such as dining in restaurants, using public transportation, and attending social gatherings, without carrying safety items like anti‐emetic medication. At follow‐up, Lara reported a key incident in which a friend vomited in her presence; she remained calm and supportive and described this as a personal milestone. She had not experienced a panic attack for several months and was not receiving any psychiatric or psychological support. She maintained her gains without ongoing interventions, including meditation or other wellness practices. Although her fear of vomiting had not fully disappeared, she reported feeling capable of tolerating uncertainty and utilizing the skills acquired during therapy. She applied cognitive restructuring for early anxiety and engaged in low‐intensity exposure for maintenance. Approximately once every two months, she used brief (7‐min) voice‐based interactions with ChatGPT‐4o in English as a distraction via general knowledge conversations. Overall, findings indicate sustained symptom reduction and meaningful functional improvement across cognitive, behavioral, and emotional domains.

## 5 Discussion

This case study contributes to the growing literature supporting the efficacy of CBT for Emetophobia with comorbid panic symptoms, while also adding to the emerging body of evidence on the clinical utility of artificial intelligence in mental health care and psychotherapy.

The therapeutic outcomes observed in this case further illustrate how core CBT techniques, when appropriately applied, can lead to substantial cognitive and behavioral changes. Consistent with core CBT principles [16], the client learned to re‐evaluate dysfunctional thoughts, resulting in reduced catastrophizing and enhanced self‐efficacy. The client developed more balanced core beliefs (e.g., “I am strong,” “I am capable of managing uncertainty,” and “I am a valuable person regardless of discomfort”) and showed meaningful behavioral gains in daily functioning.

A novel aspect of this case was the structured use of speech‐based ChatGPT in English as an attention‐shifting tool during exposure, which supported habituation by redirecting focus from threat‐based cues, particularly during out‐of‐session in vivo exposures where traditional distraction was insufficient. Although AI use could theoretically function as a safety behavior (e.g., “if anxiety arises, talking to AI will make it go away”), this did not occur, as it was integrated in a structured manner to manage attention rather than eliminate anxiety. Lara used AI alongside other strategies without exclusive reliance and reported occasional informal use for general and academic purposes, further reducing the risk of it becoming a single‐purpose safety signal. Even while interacting with AI, she continued to be exposed to vomiting‐related anxiety; therefore, although it occasionally acted as a distractor, it did not prevent exposure.

Beyond this individual case, emerging literature highlights the broader utility of AI in mental health care, including its capacity to support early detection and intervention [17, 18], and to enable continuous, remote assessment and support [19]. While promising, these results are preliminary. As a single‐case report, generalizability is limited, and further research including randomized controlled trials is needed to assess the broader clinical utility of AI‐assisted CBT. The patient's profile as a young, educated individual with proficiency in a second language likely facilitated the success of this AI‐assisted intervention; however, its efficacy may be limited for populations with different demographic or linguistic characteristics.

Future studies may explore how second‐language use interacts with emotion regulation and treatment adherence in anxiety disorders. AI‐assisted tools such as ChatGPT may serve as valuable adjuncts in exposure therapy when used with clear clinical rationale and boundaries. Psychoeducation is essential to ensure ethical use and prevent AI from becoming a safety behavior. When introduced as a structured attention‐shifting strategy rather than a substitute for CBT, AI may enhance self‐efficacy and autonomy, during challenging in vivo exposures. Clinicians should carefully assess motivation and readiness and continuously monitor its impact on avoidance, engagement, and symptom reduction. This case report may serve as a foundation for future empirical research on AI use in routine clinical practice.

## 6 Patient Perspective

In the past, even talking about vomiting would trigger anxiety. I avoided restaurants, travel, and social events. Therapy helped me face my fears step by step. Surprisingly, speaking English during anxious moments helped me stay grounded. Exposure was challenging, but I gradually felt stronger. Now, I can eat out, travel, and live more freely. I still feel fear sometimes but now, I know how to cope.

## Author Contributions

**U. Selen Kilic:** conceptualization, data curation, investigation, methodology, project administration, writing – original draft, writing – review and editing.

## Funding

The author has nothing to report.

## Ethics Statement

The participant provided written informed consent for clinical assessment, intervention, and the anonymized publication of case material, with additional consent for the use of ChatGPT. The client was informed about the tool's limitations, confidentiality considerations, and non‐clinical nature based on OpenAI's public documentation and current literature.

## Conflicts of Interest

The author declares no conflicts of interest.

## Supporting information


**Figure S1:** ccr371715‐sup‐0001‐Supplementaryfigures.docx.
**Figure S2:** ccr371715‐sup‐0001‐Supplementaryfigures.docx.
**Figure S3:** ccr371715‐sup‐0001‐Supplementaryfigures.docx.

**Table S1:** ccr371715‐sup‐0002‐Supplementarytable.docx.

## Data Availability

The raw data supporting the conclusions of this article will be made available by the authors, without undue reservation.
