# Supplementary material for: Integrating Artificial Intelligence Into Exposure Therapy: A One Year Follow‐Up Case Report of Emetophobia With Comorbid Panic Disorder
Source: Clin Case Rep. 2026 Jan 16;14(1):e71715. doi: 10.1002/ccr3.71715 (PMC12809255; doi:10.1002/ccr3.71715)
Supplement: Supplementary file 1 — Figure S1: ccr371715‐sup‐0001‐Supplementaryfigures.docx. Figure S2: ccr371715‐sup‐0001‐Supplementaryfigures.docx. Figure S3: ccr371715‐sup‐0001‐Supplementaryfigures.docx. [file CCR3-14-e71715-s001.docx]

Supplementary Figure 1

## CBT Formulation of Emetophobia and Panic Disorder

**Core and Maladaptive Beliefs**

I am vulnerable, powerless

If I feel nauseous, I won’t be able to cope

If I control my vomiting, I’ll be okay

Vomiting is fatal

I am weak and vulnerable, worthless

The world is dangerous and unpredictable

**Increased Attention Toward Potential Threats**

How nauseous do I feel?

I felt something in my stomach, I should watch it closely.

Is my heart racing?

**Increased Perception of Potential Threats**

Was that a weird sensation? I think my stomach is starting to act up.

My heart is racing, and I feel like I’m going to faint

Fight or Flight Response

**Catastrophic Thoughts and Images**

What if I vomit right now?

**Imagining choking on vomit and being unable to breathe, recalling or constructing images**

I am going to lose control, I won’t find a way out, I will be trapped.

The image of being stuck outside at night, unable to get home.

**Anxiety/ Panic Symptoms**

Heart palpitations, sweating, trembling, shortness of breath, chest tightness, dizziness

**Safety Behaviors**

carrying antihistamines, salty sticks, black bags, soda and anti-emetic medication, checking bodily sensations, overcooking food, and mentally planning for potential emergencies. Avoiding exposure to vomit, eating or smelling seafood, consuming undercooked meat or chicken, eating food prepared by others, going to hospitals or visiting hospitalized individuals, going out at night, crowded places, attending concerts or nightclubs, and traveling long distances by plane, bus, or car- particularly when using crowded public transportation.

Adapted from the CBT model described by Maack et al. (2013).

Supplementary Figure 2

2 Flowchart of AI-assisted CBT for Emetophobia and Panic Disorder

**Assessment &Diagnosis**

**Case Conceptualization**

*-continued throughout treatment-*

**Phase 1 (Sessions 1-5)**

- Psychoeducation
- Automatic thought identification
- Homework assignments
- Motivational imagery exercise

**Phase 2 (Sessions 6–10)**

- Cognitive restructuring
- Socratic questioning & reframing
- Coping cards
- Strengths-based focus

**Phase 3 (Sessions 11–33)**

- Exposure & Response Prevention

-*Interoceptive exposures* (panic symptoms)

-*Verbal, video, imaginal, in-vivo exposures* (Emetophobia symptoms)

- Response prevention (reducing safety behaviors)
- Attention-shifting strategies
- AI-assisted verbal engagement (ChatGPT)

**Follow-Up Phase**

- Symptom monitoring
- Reinforcement of cognitive and behavioral strategies
- Encouraging continued low- to moderate-intensity exposure practices.

Supplementary Figure 3

1. Outcome Measures
