# Supplementary material for: Integrating Artificial Intelligence Into Exposure Therapy: A One Year Follow‐Up Case Report of Emetophobia With Comorbid Panic Disorder
Source: Clin Case Rep. 2026 Jan 16;14(1):e71715. doi: 10.1002/ccr3.71715 (PMC12809255; doi:10.1002/ccr3.71715)
Supplement: Supplementary file 2 — Table S1: ccr371715‐sup‐0002‐Supplementarytable.docx. [file CCR3-14-e71715-s002.docx]

Supplementary Table 1

1. Exposure Hierarchy

|  | **Exposure Hierarchy** | **Exposure Type** | **Fear Rating (0-100)** |
| --- | --- | --- | --- |
|  | Going to hospital | In-Vivo | 100 |
|  | Using a crowded public transportation | In-Vivo | 90 |
|  | Being present in a social setting where alcohol is consumed | In-Vivo | 85 |
|  | Using a public transportation | In-Vivo | 80 |
|  | Watching herself vomiting | Imagery | 80 |
|  | Someone vomiting next to her | Imagery | 70 |
|  | Someone vomiting in crowd | Imagery | 65 |
|  | Watching someone vomit clearly | Video | 60 |
|  | Watching someone vomit with limited visibility | Video | 50 |
|  | Hearing vomiting | Video | 40 |
|  | Watching a cartoon character vomit | Video | 30 |
|  | Hearing the verb ‘vomit’ | Verbal | 20 |
